# SHP-2 Binds to Caveolin-1 and Regulates Src Activity via Competitive Inhibition of CSK in Response to H_2_O_2_ in Astrocytes

**DOI:** 10.1371/journal.pone.0091582

**Published:** 2014-03-14

**Authors:** Ara Jo, Hyunju Park, Sung-Hee Lee, So-Hee Ahn, Hee Ja Kim, Eun-Mi Park, Youn-Hee Choi

**Affiliations:** 1 Department of Physiology, Tissue Injury Defense Research Center, Ewha Womans University School of Medicine, Seoul, Korea; 2 Department of Pharmacology, Tissue Injury Defense Research Center, Ewha Womans University School of Medicine, Seoul, Korea; North Carolina State University, United States of America

## Abstract

Reactive oxygen species (ROS) regulate diverse cellular functions by triggering signal transduction events, such as Src and mitogen-activated protein (MAP) kinases. Here, we report the role of caveolin-1 and Src homology 2 domain-containing protein tyrosine phosphatase 2 (SHP-2) in H_2_O_2_-induced signaling pathway in brain astrocytes. H_2_O_2_-mediated oxidative stress induced phosphorylation of caveolin-1 and association between p-caveolin-1 and SHP-2. SHP-2 specifically bound to wild-type caveolin-1 similarly to c-Src tyrosine kinase (CSK), but not to phosphorylation-deficient mutant of caveolin-1 (Y14A), and interfered with complex formation between caveolin-1 and CSK. In the presence of CSK siRNA, binding between caveolin-1 and SHP-2 was enhanced by H_2_O_2_ treatment, which led to reduced Src phosphorylation at tyrosine (Tyr) 530 and enhanced Src phosphorylation at Tyr 419. In contrast, siRNA targeting of SHP-2 facilitated H_2_O_2_-mediated interaction between caveolin-1 and CSK and enhanced Src phosphorylation at Tyr 530, leading to subsequent decrease in Src downstream signaling, such as focal adhesion kinase (FAK) and extracellular signal-related kinase (ERK). Our results collectively indicate that SHP-2 alters Src kinase activity by interfering with the complex formation between CSK and phosphotyrosine caveolin-1 in the presence of H_2_O_2_, thus functions as a positive regulator in Src signaling under oxidative stress in brain astrocytes.

## Introduction

Reactive oxygen species (ROS), such as hydrogen peroxide (H_2_O_2_), superoxide (O_2_^−^), and hydroxyl radicals (OH·), are well-known regulatory signal molecules in the brain [1], [2]. Oxidative stress associated with ROS accumulation in the brain leads to the development of diverse neuropathological conditions, including Alzheimer’s disease, Parkinson’s disease, stroke, and ischemia/reperfusion injury [3], [4]. The brain consumes a large amount of O_2_ and contains high levels of transition metals, such as iron [5]. Since auto-oxidation of neurotransmitters and secretion of excitotoxic glutamate continuously occur, significant amounts of ROS are produced in the brain compared to in other organs. Moreover, neuronal membranes are enriched in polyunsaturated fatty acids and have a high ratio of membrane surface to cytoplasmic volume [6]. Due to the anatomic structure of brain cells, the extended axonal morphology, and the nonreplicating nature of neurons, the brain is more vulnerable to free radical attacks than other organs [7]. However, how brain cells survive the continuous high ROS and oxidative stress-vulnerable environment, and whether the brain has specific defense mechanisms against ROS or oxygen species are poorly understood.

Astrocytes exist in approximately 50%–90% of the brain and play a crucial role in diverse functions, including protection against metal toxicity and oxidative stress [8]–[11]. Several recent studies have demonstrated that protein phosphatase activity is increased in reactive glia following cerebral ischemia and that protein phosphatases play a neuroprotective role against oxidative stress [12]. Src homology 2 domain-containing protein tyrosine phosphatase 2 (SHP-2) (also known as PTPN11) is a tyrosine phosphatase present in the cytoplasm and highly expressed in the central nervous system (CNS) and neurons and astrocytes [13], [14]. SHP-2 reportedly protects neurons from neurodegeneration during focal ischemia/reperfusion injury [15]. Furthermore, SHP-2 inhibition lead to increased programmed cell death of primary cultured neurons in a model of CNS injury [16]. We previously showed that SHP-2 plays an immunomodulatory role against H_2_O_2_-mediated oxidative stress in brain astrocytes by regulating the activities of STAT-3 and COX-2 [9], [17]. In addition, lipid rafts and caveolin-1 are involved in astrocyte-specific intracellular responses linked to the SHP-2-mediated signaling cascade following ROS-induced oxidative stress [17], [18].

Caveolin-1, a 21–24-kDa membrane protein, is a major multifunctional scaffolding protein of caveolae that regulates a number of signaling pathways including those involved in cell migration, cell cycle, cell proliferation, cell transformation, and vesicular transport [19], [20]. In the brain, caveolin is widely expressed in astrocytes, endothelial cells, oligodendrocytes, Schwann cells, dorsal root ganglia, and hippocampal neurons [21]. Caveolin-1 is phosphorylated at Tyr 14 by Src, Abl, and Fyn in response to a variety of stimuli, including insulin, angiotensin II, osmotic shock, and oxidative stress [22]. In addition, caveolin-1 plays an essential role providing a docking site to anchor various proteins such as c-Src tyrosine kinase (Csk) in several different cell types [23], [24]. CSK is known to directly bind to caveolin-1 and suppresses Src kinase activity by inducing Src phosphorylation at the Tyr 530 residue and interfering with Src phosphorylation at Tyr 419 [22], [25]. Our recent studies have suggested that H_2_O_2_-mediated oxidative stress induces caveolin-1-SHP-2 complex formation and that caveolin-1 is involved in the ROS-induced activation of SHP-2 [18]. However, the biological significance of caveolin-1-SHP-2 complex formation and the involvement of this complex is involved in astrocyte-specific intracellular responses under oxidative stress remain unclear.

In this study, we present novel evidence and the molecular mechanisms underlying the reciprocal regulation of Src activity by SHP-2 and CSK under H_2_O_2_-mediated oxidative stress in brain astrocytes. We showed that H_2_O_2_-mediated oxidative stress induces the association between caveolin-1 and SHP-2, and that this interaction depends on the phosphotyrosine 14 residue of caveolin-1. In addition, our data show that SHP-2 alters Src activity by interfering with the complex formation between CSK and phosphotyrosine caveolin-1 in the presence of H_2_O_2_, indicating that SHP-2 functions as a positive regulator in Src signaling under oxidative stress in brain astrocytes.

## Materials and Methods

### Cells

Human astroglioma CRT-MG, U87-MG, and U251-MG cells [26], human umbilical vein endothelial cells (HUVECs), and human embryonic kidney HEK 293T cells were maintained in Dulbecco’s modified Eagle medium (DMEM; WelGENE Inc., Daegu, Korea) containing 10% fetal bovine serum (FBS, WelGENE), l-glutamine, 100 U/mL penicillin, and 10 µg/mL streptomycin in a humidified 5% CO_2_ incubator at 37°C. Primary astrocytes from the cerebral cortices of 1-day-old Sprague–Dawley rats were cultured as previously described [17]. Briefly, cortices were triturated in minimal essential medium (MEM) containing 10% FBS to yield a single-cell suspension and plated in 75-cm^2^ T-flasks (0.5 hemisphere/flask). After 2–3 weeks of culture, the flasks were shaken to detach microglia and oligodendrocytes growing on the top of the astrocytic layer. The remaining adherent astrocytes were detached using trypsin/EDTA and plated on dishes. Microglia were detached from flasks by mild shaking and filtered through a nylon mesh to remove the astrocytes. The cell identities in astrocyte- and microglia-enriched cultures were confirmed by staining for glial fibrillary acidic protein.

### Reagents and Antibodies

H_2_O_2_, N-acetyl-l-cysteine (NAC), and catalase were purchased from Sigma (St. Louis, MO, USA). PTP inhibitor IV (PTPi IV) was purchased from Calbiochem (San Diego, CA, USA). Recombinant glutathione S-transferase (GST)-SHP-2 and GST-CSK were purchased from Abnova (Taipei, Taiwan). Antibodies against caveolin-1, p-caveolin-1 (pY14) and SHP-2 were obtained from BD Transduction Laboratories (Lexington, KY, USA). Anti-CSK antibody and anti-FAK were obtained from Santa Cruz Biotechnology (Santa Cruz, CA, USA). p-SHP-2 Y542, p-SHP-2 Y580, Src, p-Src Y416, p-Src Y529, and p-FAK antibodies were obtained from Cell Signaling (Danvers, MA, USA). Tubulin antibody was obtained from Sigma. Horseradish peroxidase (HRP)-conjugated secondary antibodies for immunoblotting were obtained from Santa Cruz Biotechnology.

### Site-specific Mutagenesis of Caveolin-1 and Transient Transfection

cDNA encoding full-length human caveolin-1 was obtained from Thermo Scientific (Waltham, MA, USA). The Y14A caveolin-1 mutant was generated using a QuickChange site-directed mutagenesis kit (Stratagene, Santa Clara, CA, USA) using PCR. pCMV-SPORT6-caveolin-1 cDNA (25 ng) was used as a template and added to dNTP mix, *Pfu* buffer, *Pfu* turbo DNA polymerase and 125 ng of each of the mutagenic primers: sense, 5′-tcggagggacatctcgccaccgttcccatccg-3′ and antisense, 5′-cggatgggaacggtggcgagatgtccctccga-3′. Samples were then incubated at 95°C for 1 min, then for 20 cycles of 95°C for 1 min, 65°C for 4 min, and 68°C for 12 min. The methylated parental DNA template in the PCR product was digested using the *Dpn*I (10 U) restriction enzyme for 3 h at 37°C. The reaction mixture was then directly transformed into XL1 Blue supercompetent *Escherichia coli* cells (Stratagene) and plated onto LB agar plates containing ampicillin. DNA was extracted from selected clones using the Labopass™ Gel Extraction Kit (Cosmo Genetech Co. Ltd., Seoul, Korea) and sequenced to verify the presence of the desired mutation. After mutagenesis, 1 µg of caveolin-1 wild-type (WT) or Y14A mutant DNA was transfected into HEK 293T cells using VIVAMAGIC transfection reagent (VIVAGEN, Seongnam, Korea) containing 10% FBS according to the manufacturer’s instructions for 24 h before treatment with 5 mM H_2_O_2_ for 10 min; the cells were then analyzed by immunoblotting.

### Transfection of siRNA

Cells were plated and transfected with green fluorescence protein (GFP) siRNA (Samchully Pharm Co. Ltd., Seoul, Korea) as a control group and human CSK siRNA and human SHP-2 siRNA from Bioneer (Daejeon, Korea) using Lipofectamine RNAiMAX (Invitrogen, Carlsbad, CA, USA) according to the manufacturer’s instructions to achieve a final concentration of 300 nM. Cells were allowed to recover in DMEM containing 1% FBS for 48 h before treatment with 5 mM H_2_O_2_ for 10 min and were then analyzed by immunoblotting.

### Co-immunoprecipitation

Cells were washed with phosphate-buffered saline (PBS) followed by lysis in cold radioimmunoprecipitation assay (RIPA) buffer with protease inhibitors (1% Nonidet-P 40, 0.5% sodium deoxycholate, 10 mM disodium hydrogen phosphate, 150 mM sodium chloride, 1 mM EDTA, 0.1% SDS, 0.5 mM sodium orthovanadate, 10 µg/mL aprotinin, 10 µg/mL leupeptin, and 1 mM phenylmethylsulfonyl fluoride) for 1 h on ice. Total lysates (700 µg) were pre-cleared by incubating with protein A-agarose beads (Biovision Inc., Mountain View, CA, USA) for 3 h at 4°C. After centrifugation, cleared lysates were incubated with 1 µg of anti-caveolin-1, anti-SHP-2, and anti-CSK antibodies at 4°C overnight, and precipitated with protein A-agarose beads for 3 h at 4°C. Immunoprecipitated proteins were separated by 10% or 12% SDS-PAGE, and input proteins were analyzed by immunoblotting as described.

### Immunoblot Analysis

Cells were lysed in ice-cold RIPA buffer containing protease inhibitors for 1 h on ice. Lysates were centrifuged (12,000×*g*) at 4°C for 30 min and resolved by 10% or 12% SDS-PAGE. Separated proteins were transferred to polyvinylidene difluoride membranes (Bio-Rad, Hercules, CA, USA), and then blots were probed with caveolin-1, p-caveolin-1, SHP-2, p-SHP-2, CSK, Src, p-Src, ERK, p-ERK, FAK, p-FAK and tubulin antibodies. After incubating with specific secondary antibodies, the blots were exposed to blue X-ray film (AGFA, Mortsel, Belgium) by using an enhanced chemiluminescence system (Amersham, Buckinghamshire, UK).

### *In situ* Proximity Ligation Assay (PLA)

Protein-protein interactions were detected using the Duolink II *in situ* proximity ligation assay (PLA) Kit (Olink Bioscience, Uppsala, Sweden). Cells were plated and treated with or without 5 mM H_2_O_2_ for 10 min, fixed with 100% methanol at −20°C for 30 min, and then incubated overnight with antibodies against caveolin-1 and SHP-2 at 1∶100 dilution. After washing, the PLA probe PLUS against SHP-2 and PLA probemaker probe against caveolin-1 were incubated for 3 h at 37°C and the ligases (1 U/µL) diluted at 1∶40 were incubated for 2 h at 37°C. Circular oligonucleotides were amplified using polymerase (10 U/µL) diluted at 1∶80 for 100 min at 37°C. Samples were viewed using a Carl Zeiss confocal microscope (Carl Zeiss AG, Oberköchen, Germany). Nuclei were stained with VECTASHIELD HardSet™ mounting medium with DAPI (Vector Laboratories Inc., Burlingame, CA, USA). Experiments were repeated 4 times and showed similar results.

### PepSPOTs Assay

Identification of binding sites for recombinant SHP-2 and CSK in the caveolin-1 protein was performed using PepSPOTs (JPT Peptide Technologies GmbH, Berlin, Germany). Synthetic peptide sequences of caveolin-1 (1–85 and 1–28) were used in this study. The peptide library contained either 13-mer peptides that overlapped by 5 residues or 13-mer peptides that overlapped by 8 residues. Each spot carried approximately 5 nmol of peptide covalently bound to the cellulose-betaalanine-membrane (N-terminus: acetyl). Two identical membranes were incubated overnight with either recombinant GST-SHP-2 (rGST-SHP-2) or GST-CSK (rGST-CSK) respectively, and immunoblotting was performed to detect rGST-SHP-2/caveolin-1 peptide and rGST-CSK/caveolin-1 peptide interactions.

### Surface Plasmon Resonance (SPR)

SPR was performed on a SPRmicro system (K-MAC, Daejeon, Korea). A G1 chip (bare surface gold chip) was dipped into a 3 mM N-hydroxylsuccinimide self-assembled monolayer to form covalent bonds among protein amine groups. After washing with ethanol, the chip was purged with N_2_ gas. Recombinant GST-SHP-2 or GST-CSK was mixed with 20% ethylene glycol and then spotted onto the chip. Ligands were reacted on room temperature for 12 h to allow the ligands to become fixed onto the chip. After the reaction, the ligands were removed by washing with 1×Tris-buffered saline (TBS), and purged with N_2_ gas. SPR signal baselines were stabilized using 1×TBS running buffer, and different dilutions of caveolin-1 phosphopeptides (Cav-1 pY 14, GHL-pY-TVPIREQGN) in buffer were injected at a flow rate of 20 µL/min. Response unit (RU) alterations following sample reactions were analyzed by comparing affinities to the chip surface. Experiments were conducted by switching from samples immobilized on the chip (Cav-1 pY 14) to injected samples (rGST-CSK or rGST-SHP-2). Experiments were repeated twice and showed similar results. Equilibrium binding curves and RU values are shown.

### Protein Expression and Purification

Human SHP-2 and CSK fusion constructs were produced by consecutively subcloning the protein coding regions of SHP-2 and CSK into the pGEX-6P-1 vector (GE healthcare, Piscataway, NJ, USA). Using these constructs, human SHP-2 and CSK were overexpressed in *Escherichia coli* (BL21) as soluble. Briefly, the transformed bacteria were grown in LB medium with 0.1 mg/ml ampicillin at 37°C to an A600 of 0.6, and then cultured for an additional overnight at 27°C after being induced with 0.5 mM IPTG (Duchefa Biochemie, Netherlands). The cells were harvested by centrifugation at 8000 *g* for 20 min, resuspended in PBS pH 7.4, and then disrupted by ultrasonication. The supernatant was purified with glutathione agarose 4B (Incospharm, Daejeon, South Korea) in PBS pH 7.4. The bound proteins were eluted with a linear gradient 10 mM glutathione in 50 mM Tris (pH 8.0). All proteins were concentrated with a Centricon apparatus (Miliipore, Darmstadt, Germany). Proteins were quantitated with the Bradford assay, then filtered and stored at −70°C until use.

### Flow Cytometry

Conjugation of 1–50 mer caveolin-1 peptide (PEPTRON, Daejeon, South Korea) to carboxyl beads (Bangs Laboratories, Fishers, IN, USA) using 1-Ethyl-3-(3-dimethylaminopropyl) carbodiimide (EDC) [27]. After washing the carboxyl beads with distilled water (DW), the carboxyl beads were incubated with caveolin-1 peptide 25 µg/ml (1–50) and 1% EDC (Pierce Biotechnology, Rockford, IL, USA) for overnight at 37°C with shaking. Then we add 10 mM of glycine and 10 mM glucose for 30 min at RT. Caveolin-1 peptide Conjugated beads were incubated with recombinant CSK or SHP-2 protein for 1 h at RT with shaking. After wash with PBS, beads were incubated with 1 µg of anti-CSK or anti-SHP-2 antibodies at 4°C for 1 h. The beads were then incubated with FITC-conjugated goat anti-rabbit IgG (Southern Biotech, Birmingham, AL, USA) or Alexa 488-conjugated goat anti-mouse IgG (Life Technologies, Grand Island, NY, USA) for 1 h and washed with PBS. Flow cytometric measurements were carried out on Accuri C6 flow cytometer (BD Bioscience, San Jose, CA, USA). Experiments were repeated twice and showed similar results.

### Statistical Analysis

Statistical analyses were performed using the Student’s *t* test to compare between sample groups, and ANOVA was used to determine differences among multiple groups. Statistical significance of the data was set at *P*<0.05.

## Results

### H_2_O_2_ Induces Caveolin-1 Phosphorylation at Tyr 14 in Brain Astrocytes

**Figure 1:**
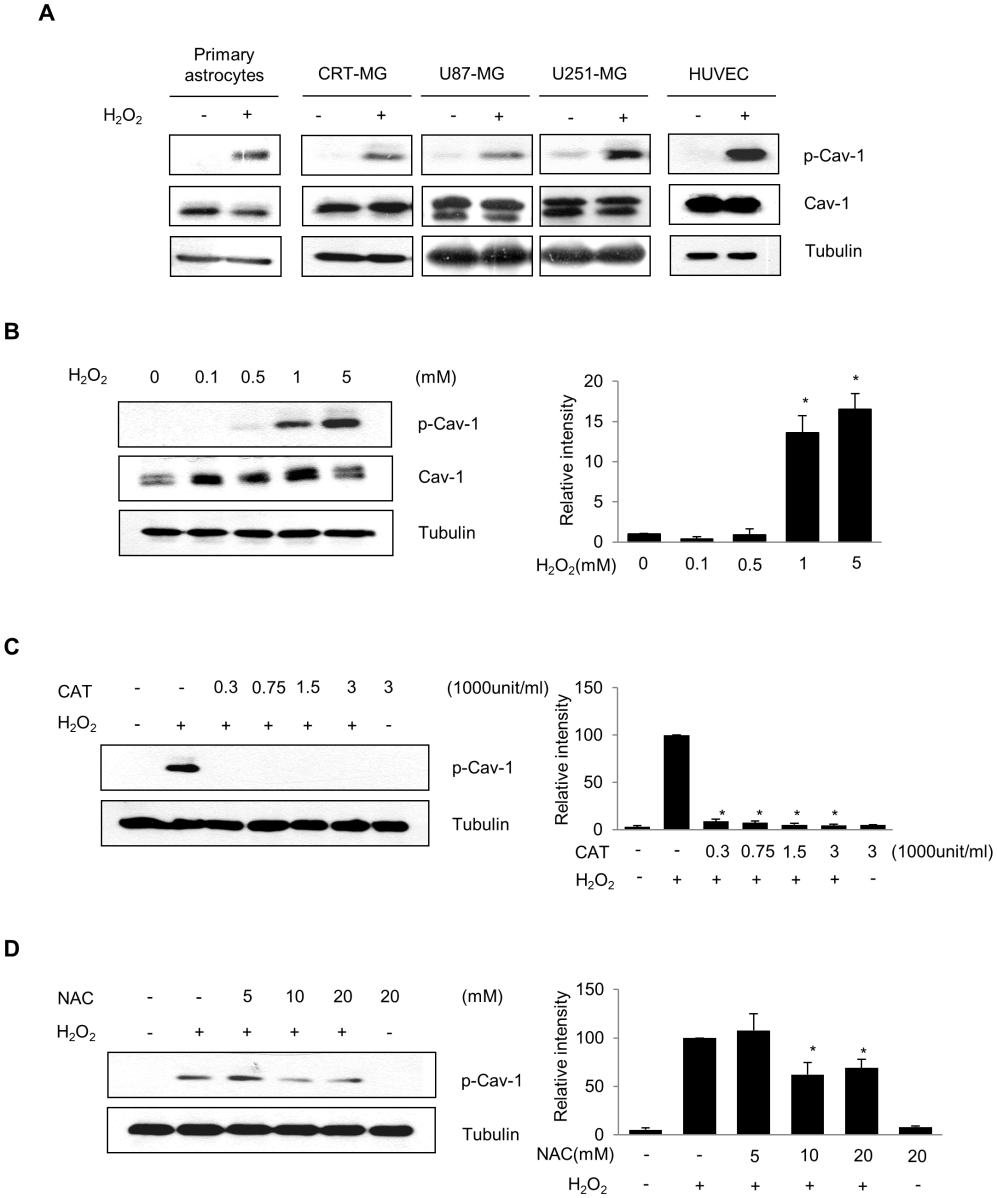
H_2_O_2_ induces caveolin-1 phosphorylation at Tyr 14 in rat primary astrocytes and human astroglioma cells. (A) Rat primary astrocytes, human astroglioma CRT-MG, U87-MG, and U251-MG, and human umbilical vein endothelial cells (HUVECs) were plated and treated with 1 mM H_2_O_2_ for 10 min, and then whole-cell lysates (WCLs) in cold-RIPA buffer were extracted and subjected to immunoblotting analysis. Blots were probed with antibodies against p-caveolin-1, caveolin-1, and tubulin. HUVECs were utilized as a positive control. Data are representative of at least three experiments. (B) CRT-MG cells were plated and incubated with a range of H_2_O_2_ concentrations (0–5 mM) for 10 min, and WCLs were extracted using cold RIPA buffer. Antibodies against caveolin-1, p-caveolin-1, and tubulin were used for immunoblotting. Right panel, Densitometric quantification of p-caveolin-1 level was normalized to the level of tubulin. Values are mean ± SD from three experiments. *p<0.05, versus untreated control. (C, D) CRT-MG cells were pretreated with various concentrations of catalase (unit/mL) (C) or NAC (mM) (D) as indicated in the figure for 30 min, and 5 mM H_2_O_2_ for 10 min. The cells were analyzed by immunoblotting with both anti-p-caveolin-1 and anti-tubulin antibodies. Right panel, Densitometric quantification of p-caveolin-1 protein levels, normalized to the level of tubulin. Data are expressed as percentage of H_2_O_2_-treated values presented as mean ± SD. (CAT, catalase; Cav-1, caveolin-1; p, phospho). *p<0.05, versus H_2_O_2_-treated cells. Data are representative of three independent experiments.

Previous studies demonstrated that H_2_O_2_ induces the phosphorylation of caveolin-1 at Tyr 14 in bovine pulmonary artery endothelial cells and placenta artery endothelial cells [28]–[30]. To determine whether H_2_O_2_ induces the phosphorylation of caveolin-1 in brain astrocytes, rat primary astrocytes, human astroglioma cells (CRT-MG, U87-MG, and U251-MG) and HUVECs were treated with 1 mM H_2_O_2_ for 10 min and examined by immunoblotting for p-caveolin-1. H_2_O_2_ induced the phosphorylation of caveolin-1 in the presence of H_2_O_2_ in primary astrocytes, astroglioma cells, and endothelial cells (Fig. 1A). H_2_O_2_ induced a dose-dependent increase in the level of Tyr-phosphorylated caveolin-1 up to a concentration of 5 mM in CRT-MG cells, with no change in the amount of caveolin-1 or tubulin (Fig. 1B). To determine whether H_2_O_2_ is directly involved in caveolin-1 phosphorylation at Tyr 14, cells were treated with catalase and the potent ROS scavenger NAC. Both catalase and NAC considerably suppressed H_2_O_2_-induced caveolin-1 phosphorylation in a dose-dependent manner (Fig. 1C and 1D). These results indicate that Tyr 14 of caveolin-1 is phosphorylated in response to H_2_O_2_ in brain astroglioma cells, which agrees with the results of previous studies using bovine pulmonary artery endothelial cells and placenta artery endothelial cells.

### H_2_O_2_-mediated Association of CSK and Caveolin-1 is Dependent on the Phosphorylation of Caveolin-1 at Tyr 14 in Human Astroglioma Cells

**Figure 2:**
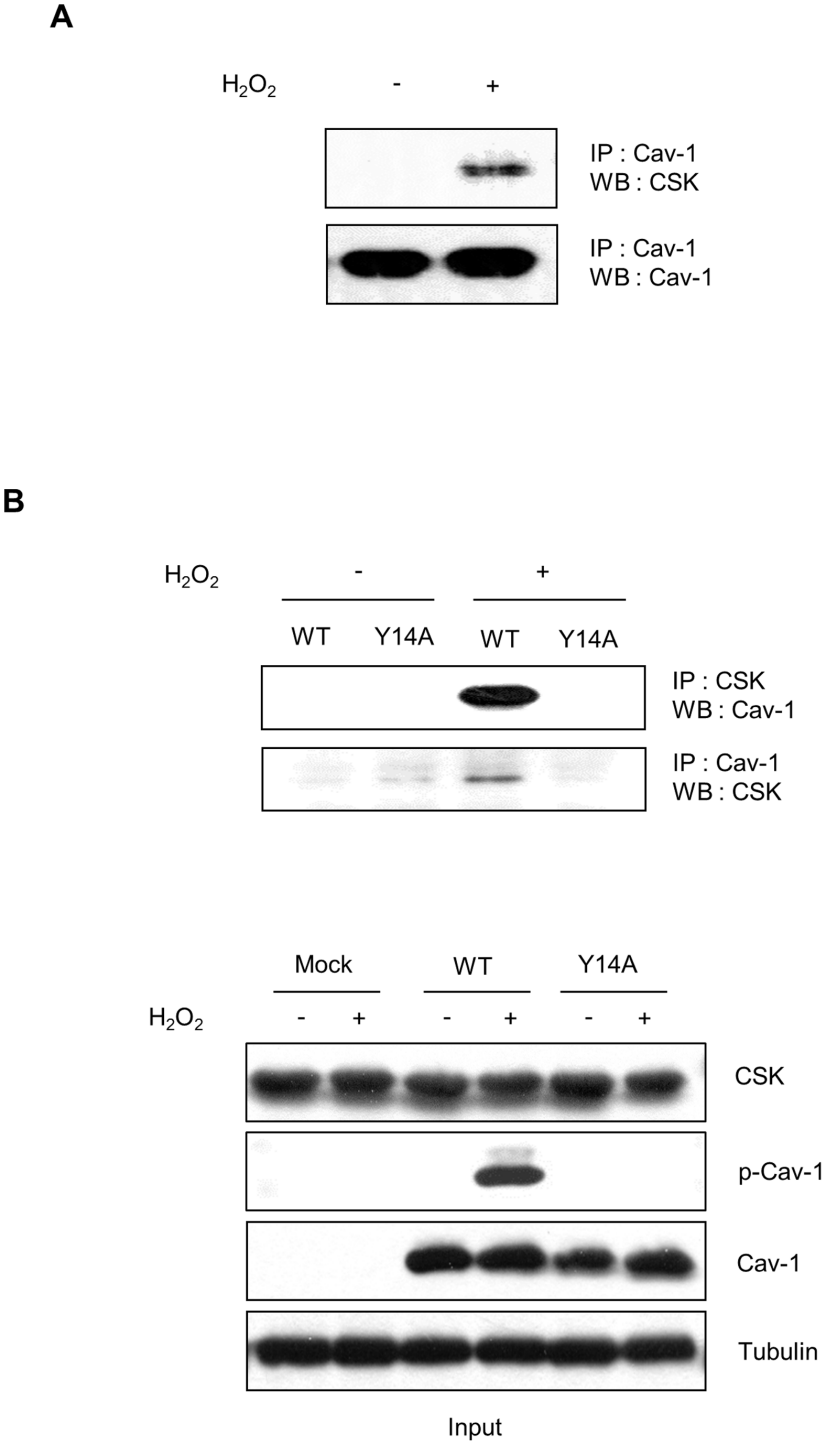
H_2_O_2_-mediated association of CSK and caveolin-1 is dependent on the phosphorylation of caveolin-1 at Tyr 14. (A) CRT-MG cells were treated with or without H_2_O_2_ for 10 min, and whole-cell lysates (WCLs) were extracted. A total of 700 µg of WCL was immunoprecipitated with an anti-caveolin-1 antibody. Immunoprecipitates were analyzed by immunoblotting with anti-CSK and anti-caveolin-1 antibodies. (B) Human embryonic kidney cell line HEK 293T cells were transfected with empty vector (Mock), wild-type (WT) and mutant caveolin-1 (Y14A). After 24 h, cells were treated with 5 mM H_2_O_2_ for 10 min or left untreated and lysed in RIPA buffer. WCLs (700 µg) were precleared and immunoprecipitated with anti-CSK and anti-caveolin-1 antibodies and then analyzed by immunoblotting. Input (5%) is shown (Cav-1, caveolin-1 and pY14-caveolin-1). Data are representative of at least 3 experiments.

Caveolin-1 has been implicated as a CSK adapter and several reports demonstrated that CSK interacted only with Tyr 14-phosphorylated caveolin in response to VEGF, angiotensin II, insulin, v-Abl, EGF, and oxidative stress [22], [31], [32]. To confirm whether CSK interacts with Tyr 14-phosphorylated caveolin in response to H_2_O_2_ in astrocytes, a co-immunoprecipitation assay was performed using total lysates from CRT-MG cells. As shown in Fig. 2A, complex formation occurred between caveolin-1 and CSK following H_2_O_2_ treatment of CRT-MG cells. To explore the role of the caveolin-1 Tyr 14 residue in the interaction between caveolin-1 and CSK, HEK 293T cells were used because they are known to express caveolin-1 at a very low level. The cells were transfected with plasmids encoding either WT caveolin-1 or a phosphorylation-deficient caveolin-1 mutant (Y14A), treated with or without H_2_O_2_, and then co-immunoprecipitated with either anti-CSK or anti-caveolin-1 antibodies. An interaction between CSK and WT caveolin-1, but not Y14A mutant caveolin-1, was observed in the presence of H_2_O_2_ (Fig. 2B). These results indicate that H_2_O_2_-induced formation of the caveolin-1/CSK complex is dependent on the Tyr 14 residue of caveolin-1.

### H_2_O_2_ Induces SHP-2-caveolin-1 complex Formation in Human Astroglioma Cells

**Figure 3:**
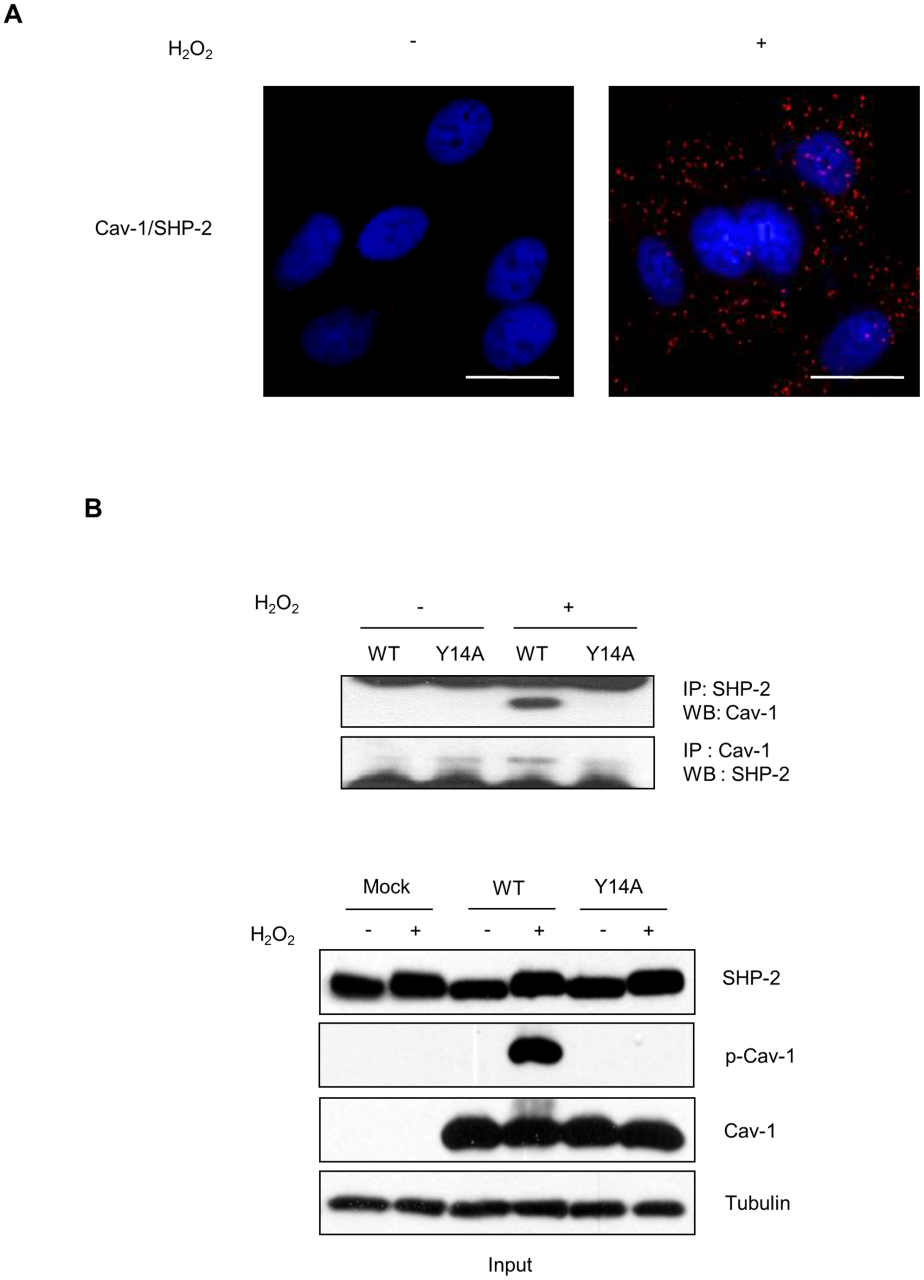
H_2_O_2_ induces SHP-2-caveolin-1 complex formation. (A) Complex formation between caveolin-1 and SHP-2 in CRT-MG cells as investigated by *in situ* proximity ligation assay (PLA). CRT-MG cells were incubated for 10 min with 5 mM H_2_O_2_, fixed, and then incubated overnight with antibodies against caveolin-1 and SHP-2. After washing, the PLA probe PLUS and PLA Probemaker probe were incubated for 3 h at 37°C and diluted ligases at 1∶40 were incubated for 2 h at 37°C. Circular oligonucleotides were amplified using polymerase for 100 min at 37°C. Nuclei were stained with DAPI. The experiments were repeated 4 times with similar results. Bars = 20 µm. (B) pCMV-SPORT6, Caveolin-1 WT or caveolin-1 Y14A DNA were transiently transfected into HEK 293T cells. Next, 24 h after transfection, cells were incubated with 5 mM H_2_O_2_ for 10 min. WCLs (700 µg) were subjected for immunoprecipitation using the anti-caveolin-1 and anti-SHP-2 antibodies and analyzed by immunoblotting. Input (5%) is shown. Data are representative of at least 3 experiments.

We previously showed that SHP-2 is translocated into lipid rafts and activated in response to H_2_O_2_, while caveolin-1 is involved in ROS-induced SHP-2 activation through protein-protein interactions in brain astrocytes [17]. To confirm H_2_O_2_-induced complex formation between SHP-2 and caveolin-1, an *in situ* PLA, which enables the detection of direct protein-protein interactions between 2 proteins by visualization *in situ* as a fluorescent red dot [33], was performed using CRT-MG astroglioma cells. Images obtained from the PLA showed that a direct interaction between SHP-2 and caveolin-1 (indicated by red dots) occurred in the presence of H_2_O_2_ in CRT-MG cells (Fig. 3A). To test whether this H_2_O_2_-induced interaction between SHP-2 and caveolin-1 depends on the Tyr 14 residue of caveolin-1, similar to the interaction between CSK and caveolin-1, a co-immunoprecipitation assay was performed in HEK 293T cells that were transfected with either WT or Y14A caveolin-1 DNA in the absence or presence of H_2_O_2_. As shown in Fig. 3B, SHP-2 interacted with WT caveolin-1, but not mutant caveolin-1, and only in the presence of H_2_O_2_. These results indicate that SHP-2 forms a complex with caveolin-1 in response to H_2_O_2_ in brain astrocytes and that H_2_O_2_-induced caveolin-1 and SHP-2 complex formation is dependent on the Tyr 14 residue of caveolin-1.

### Both SHP-2 and CSK Specifically Bind to Tyr-phosphorylated Caveolin-1 (Tyr 14)

**Figure 4:**
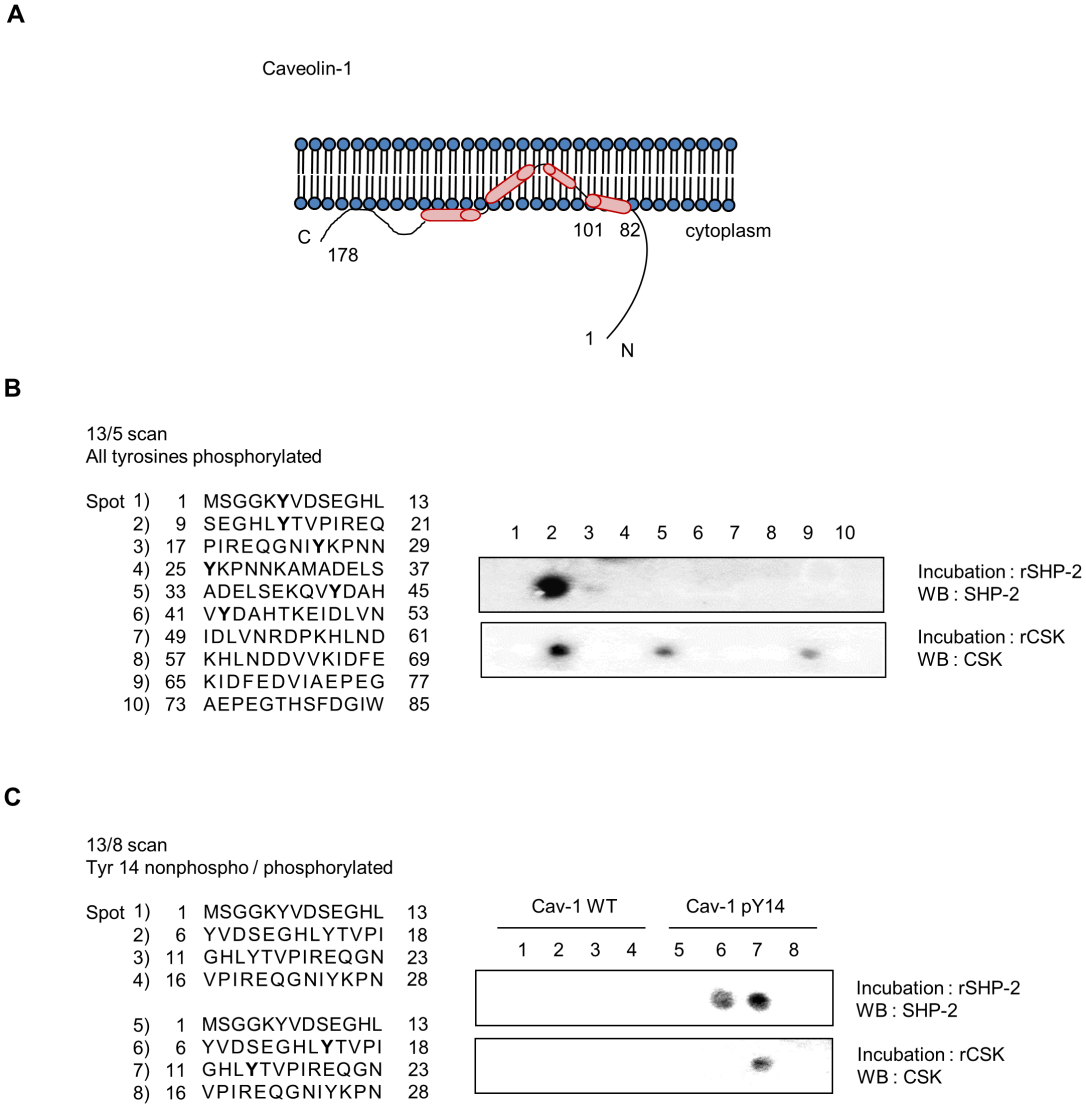
Both SHP-2 and CSK bind specifically to phosphorylated caveolin-1 at Tyr 14. (A) Schematic diagram of caveolin-1. (B) A peptide array assay was performed to identify the binding site for recombinant SHP-2 and CSK in the caveolin-1 protein. Schematic diagram of the synthetic peptide sequences of caveolin-1 (1–85) spotted on the membrane used for study is shown in the left panel. The peptide numbering starts with 1 in the left corner and the phosphorylated tyrosine residues are indicated in bold. Two identical membranes were incubated overnight with recombinant SHP-2 and CSK protein and immunoblotting was performed to detect SHP-2/caveolin-1 and CSK/caveolin-1 interactions. The experiments were repeated 3 times and showed similar results. (C) Schematic diagram of synthetic peptide sequences of caveolin-1 (1–28) used in this study is shown in the left panel. The peptide numbering starts with 1 in the left corner. Phosphorylated Tyr 14 is indicated in bold. Membranes were incubated overnight with recombinant SHP-2 and CSK and immunoblotting was performed as described in B (Cav-1, caveolin-1; WT, wild-type; pY 14, phospho-tyrosine 14). Data are representative of at least 3 experiments.

Since both SHP-2 and CSK bound to caveolin-1 by recognizing the specific phosphorylated Tyr 14 residue, other tyrosine residues in caveolin-1 were examined to determine if they also mediate binding to SHP-2 or CSK. Thirteen-amino acid-long synthetic peptides of caveolin-1 on PepSPOT were used for binding analysis. Four Tyr residues (Tyr 6, Tyr 14, Tyr 25, and Tyr 42) were phosphorylated (marked in bold, Fig. 4B, left). Duplicate membranes containing bound caveolin-1 peptides were incubated with either 1 µg recombinant GST-SHP-2 (rGST-SHP-2) or GST-CSK (rGST-CSK) and washed, and the binding of SHP-2 or CSK to the caveolin-1 peptides was analyzed by immunoblotting. PepSPOT analysis showed that only 1 spot of bound recombinant SHP-2 was detected, corresponding to residues 9–21 of caveolin-1 (spot 2), which included Tyr 14. Similar to SHP-2, strong binding between caveolin-1 peptides and recombinant CSK was detected in spot 2; however, weak binding was also observed in spots 5 and 9. Based on these experiments with pairs of synthetic nonphosphorylated-Tyr and corresponding phosphorylated-Tyr 13-mer peptides of caveolin-1 (1–28) spotted on the membrane, strong binding of both rGST-SHP-2 and rGST-CSK to caveolin-1 was observed only in spots containing phosphorylated Tyr 14 residues (Fig. 4C).

### SPR Analyses of SHP-2 and CSK Binding to Phosphorylated-Tyr Peptides of Caveolin-1

**Figure 5:**
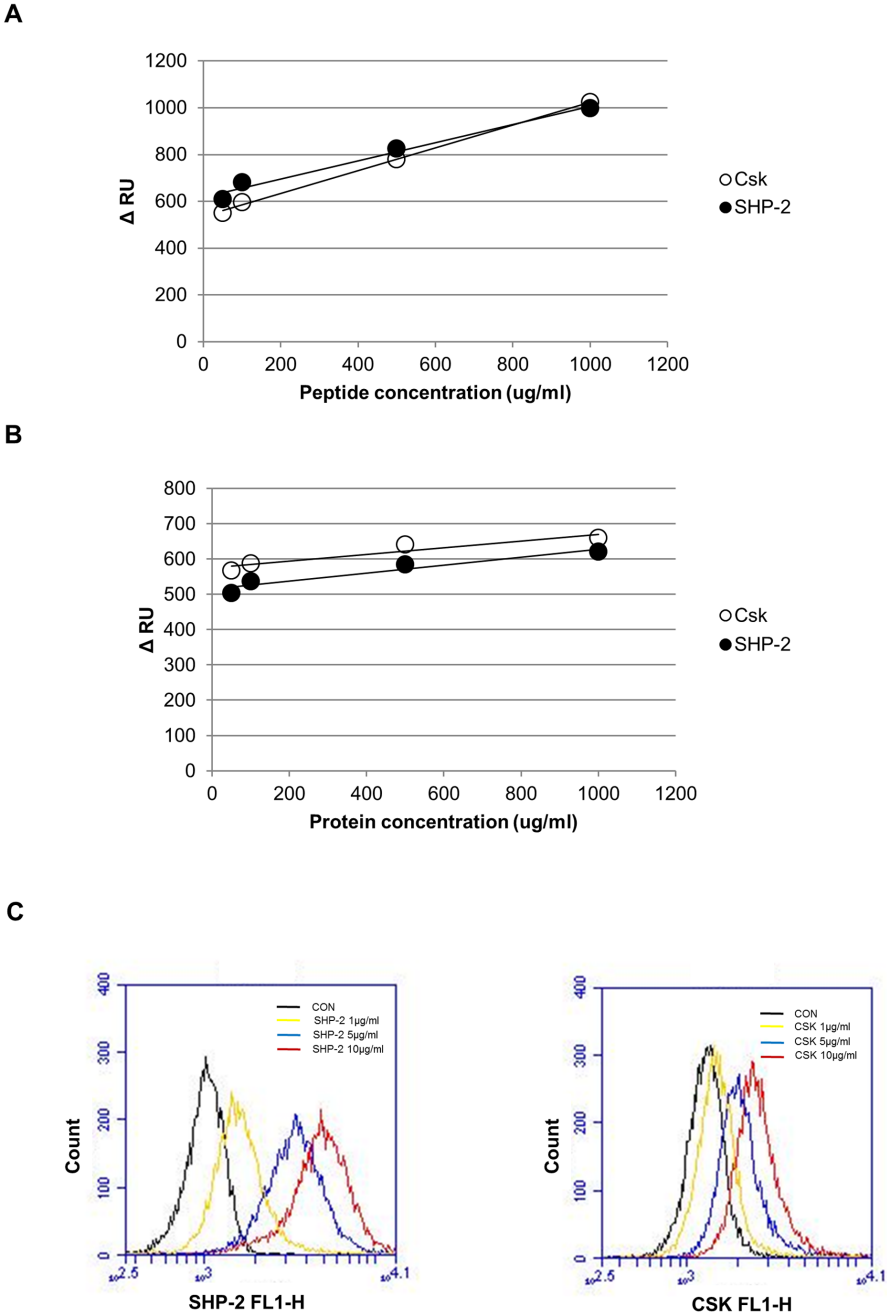
SPR and flow cytometric analyses of SHP-2 and CSK binding to phosphorylated-Tyr peptides of caveolin-1. (A) Surface plasmon resonance (SPR) was used to assess binding of either CSK or SHP-2 to the caveolin-1 peptides containing pY14. Recombinant GST-SHP-2 (closed circle) or GST-CSK (open circle) was immobilized on the sensor chip and Cav-1 pY14 was injected. Equilibrium binding curves and the response or resonance unit (RU) are shown. (B) Cav-1 pY 14 were immobilized via covalently bound streptavidin on the sensor chip. Recombinant GST-SHP-2 or GST-CSK was injected at the indicated concentrations, and the equilibrium binding curves and RU are shown. (C) Flow cytometric binding assay was performed by incubating caveolin-1 peptide conjugated beads with recombinant CSK or SHP-2 protein (1–10 µg). After wash with PBS, beads were incubated with 1 µg of anti-CSK or anti-SHP-2 antibodies and then incubated with FITC- or Alexa 488-conjugated IgG antibodies as described in Materials and Methods. After stringent wash, bound SHP-2 or CSK to caveolin-1 peptide conjugated beads were measured by Accuri C6 flow cytometer. Experiments were repeated twice with showed similar results.

To confirm that direct interaction occurs between SHP-2 and caveolin-1 and to compare the binding affinity of SHP-2 and CSK to phosphorylated-Tyr caveolin-1, SPR experiments were performed using a SPRmicro system (K-MAC, Daejeon, Korea) as described in Materials and Methods. Real-time association kinetics of either recombinant GST-SHP-2 (rGST-SHP-2) or rGST-CSK immobilized on a G1 chip with a synthetic peptide phosphorylated at a Tyr residue corresponding to Tyr 14 of caveolin-1 (Cav-1 pY 14, GHL-pY-TVPIREQGN) were analyzed by increasing peptide concentrations from 10 to 1,000 µg/mL. As shown in Fig. 5A, both rGST-SHP-2 and rGST-CSK were found to bind to Cav-1 pY 14 with high affinity and there was no significant difference in their kinetic rates or RUs. Reverse-SPR experiments were performed by immobilizing Cav-1 pY 14 on the chip and using rGST-SHP-2 or rGST-CSK as a probe (Fig. 5B); the results were similar to those observed in Fig. 5A. Next, we performed bead-based flow cytometric binding assay to confirm the direct interaction between SHP-2 and CSK to p-caveolin-1. Fifty-amino acid-long synthetic peptides of caveolin-1 (1–50 a.a.) were conjugated to carboxyl beads using EDC as described in Materials and Methods. Caveolin-1 peptide conjugated beads were incubated with either CSK or SHP-2 protein (1–10 µg), anti-CSK, or anti-SHP-2 antibodies and then incubated with FITC- or Alexa 488-conjugated IgG antibodies. As shown in Fig. 5C, both SHP-2 and CSK significantly bound to p-caveolin-1 peptide, in consistent to PepSPOTs assay (Fig. 4) and SPR analyses.

### siRNA Targeting CSK Facilitates H_2_O_2_-mediated Binding between Caveolin-1 and SHP-2

**Figure 6:**
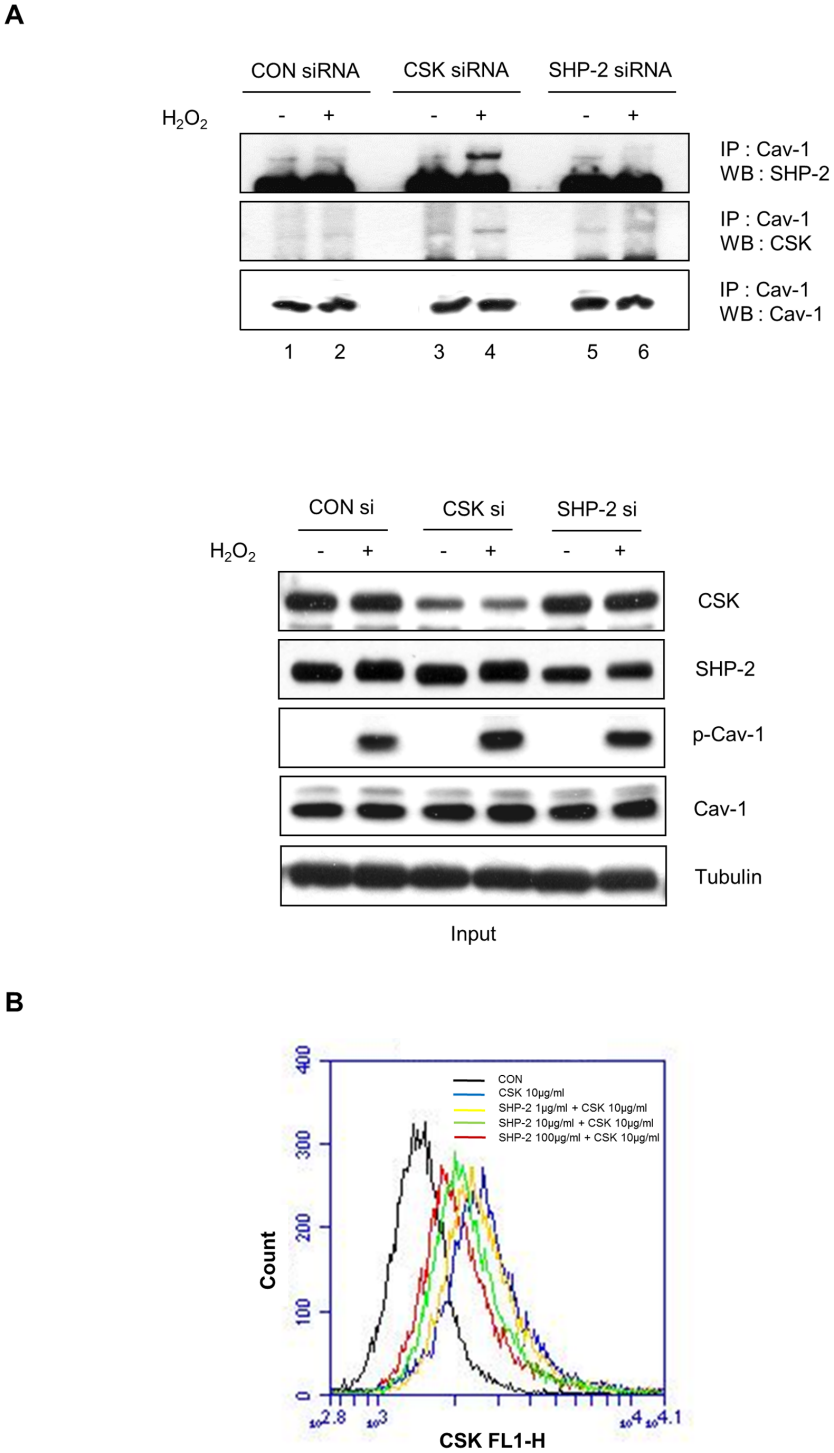
Small interfering RNA targeting CSK facilitates H_2_O_2_-mediated binding between caveolin-1 and SHP-2. (A) CRT-MG cells were transfected with GFP siRNA (Con siRNA), CSK siRNA, and SHP-2 siRNA. At 48 h after transfection, the cells were treated with 5 mM H_2_O_2_ for 10 min and lysed in RIPA buffer. Whole-cell lysates (700 µg) were subjected to immunoprecipitation with anti-caveolin-1 antibody and immunoblot with either anti-SHP-2, anti-CSK, or anti-caveolin-1 antibodies. Input (5%) is shown. (B) Flow cytometric competitive binding assay. Caveolin-1 peptide conjugated beads were incubated with 10 µg of recombinant CSK protein with or without SHP-2 protein (1–100 µg). After wash with PBS, beads were incubated with 1 µg of anti- CSK antibody at 4°C for 1 h, and then incubated with FITC-conjugated goat anti-rabbit IgG antibody for additional 1 h. Fluorescent intensities were measured by Accuri C6 flow cytometer. Experiments were repeated twice and showed similar results.

Since we found that both SHP-2 and CSK directly bind to caveolin-1, we next examined whether SHP-2 and CSK compete with each other for binding to caveolin-1 by performing SHP-2 and CSK depletion using siRNA and co-immunoprecipitation assays in the presence or absence of H_2_O_2_. In the presence of CSK siRNA, the association between caveolin-1 and SHP-2 was dramatically increased following H_2_O_2_ treatment compared to that of the control group (Fig. 6, upper panel, compare lanes 4 and 2). In addition, in the presence of SHP-2 siRNA, H_2_O_2_-induced formation of caveolin-1/CSK complexes was slightly enhanced compared to that of the control (Fig. 6, lower panel, compare lanes 6 and 2). To confirm whether SHP-2 competes with CSK for binding to p-caveolin-1, flow cytometric competitive binding assay was performed. Caveolin-1 peptide conjugated beads were incubated with 10 µg of recombinant CSK protein with or without SHP-2 protein (1–100 µg). After wash, beads were incubated with anti-CSK antibody and FITC-conjugated goat anti-rabbit IgG antibody, and then analyzed by flow cytometry. The amount of bound CSK on the p-caveolin-1 peptide conjugated beads was reduced by adding increasing concentrations of SHP-2 protein (1–100 µg). These findings indicate that knockdown of CSK facilitates H_2_O_2_-induced complex formation between caveolin-1 and SHP-2 and that reciprocal binding of SHP-2 and CSK to caveolin-1 occurred in H_2_O_2_-stimulated astrocytes.

### SHP-2 and CSK Reciprocally Regulate H_2_O_2_-induced Src Activation

**Figure 7:**
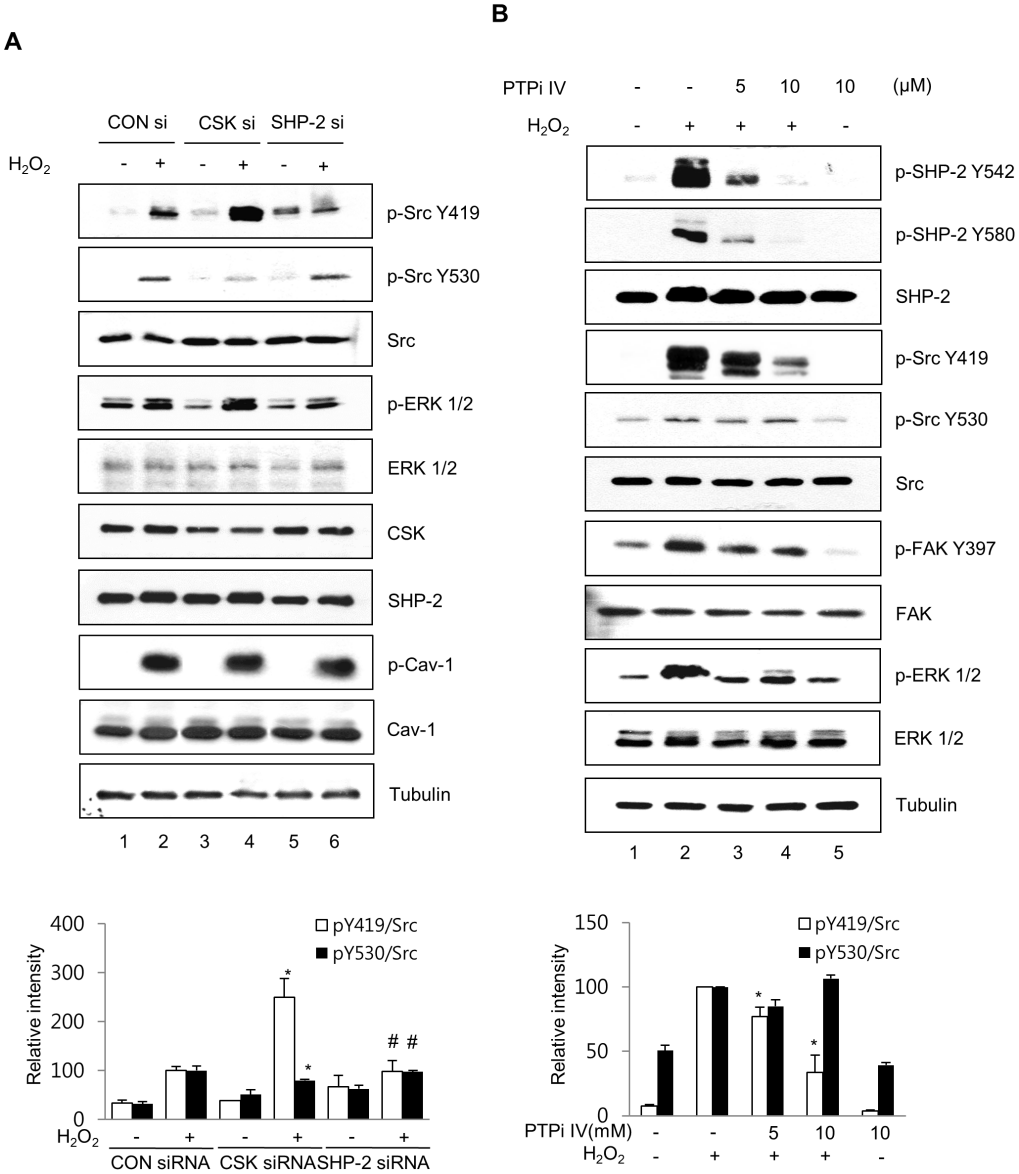
SHP-2 and CSK reciprocally regulate H_2_O_2_-induced Src activation. (A) CRT-MG cells transfected with GFP siRNA, CSK siRNA, and SHP-2 siRNA for 48 h were treated with 5 mM H_2_O_2_ for 10 min and whole-cell lysates were extracted and immunoblotted using an anti-p-Src Y419, anti-p-Src Y530, anti-Src, anti-pERK1/2, anti-ERK, anti-CSK, anti-SHP-2, anti-caveolin-1, and anti-p-caveolin-1 antibodies. Tubulin was evaluated as a loading control. Bottom panel, Densitometric quantification of p-Src Y419 levels (empty bars) and p-Src Y530 levels (filled bars), normalized to the level of total Src. Results are expressed as percentage of H_2_O_2_-treated values in control siRNA presented as mean ± SD. (*p<0.05 vs. H_2_O_2_-treated cells transfected with control siRNA; #*p*<0.05 between SHP-2 siRNA-transfected cells and CSK siRNA-transfected cells; *n* = 3). (B) Inhibition of SHP-2 by PTPi IV attenuated the H_2_O_2_-induced activation of the Src signaling pathway. CRT-MG cells were treated with 5 mM H_2_O_2_ or not for 10 min in the presence of 5 or 10 µM PTPi IV for 30 min, and lysed in cold-RIPA buffer. Antibodies against p-SHP-2, SHP-2, p-Src, Src, p-FAK, FAK, p-ERK, ERK, and tubulin were used for immunoblotting. Bottom panel, Densitometric quantification of p-Src Y419 levels (empty bars) and p-Src Y530 levels (filled bars), normalized to the level of total Src. Data are expressed as percentage of H_2_O_2_-treated values presented as mean ± SD (*n* = 3). *p<0.05 vs. H_2_O_2_-treated cells. (p, phospho).

CSK is known to directly bind to caveolin-1 and suppress c-Src kinase activity by inducing c-Src phosphorylation at Try 530 [22]. To test whether specific siRNA-mediated depletion of CSK or SHP-2 under oxidative stress affects Src activity, CRT-MG cells were transfected with CSK or SHP-2 siRNA for 48 h, treated with or without H_2_O_2_ for 10 min, and immunoblotted with anti-Src, anti-p-Src Y416 (Y419 in human), anti-p-Src Y27 (Y530 in human), anti-CSK, anti-SHP-2, anti-caveolin-1, anti-p-caveolin-1 and anti-tubulin antibodies. In the presence of CSK siRNA, the phosphorylation level of Src Y530 decreased following H_2_O_2_ treatment (Fig. 7A, second panel, compare lanes 4 and 2), while the phosphorylation level of Src Y419 was significantly increased under H_2_O_2_ treatment compared to that of the GFP siRNA-transfected cells (CON siRNA; first panel, compare lanes 4 and 2). In contrast, in the presence of SHP-2 siRNA, the level of p-Src Y530 activity upon H_2_O_2_ treatment was comparable to that of the control (Fig. 7A, second panel, compare lanes 6 and 2); however, p-Src Y419 phosphorylation was significantly reduced following H_2_O_2_ treatment compared to that of the CSK and GFP siRNA groups (first panel, compare lanes 6 and 2, 4). To examine the effect of siRNA-mediated depletion of CSK or SHP-2 on Src-downstream signaling in response to H_2_O_2_, the same lysates were analyzed by immunoblotting against p-ERK. In the presence of CSK siRNA, the phosphorylation level of ERK was increased by H_2_O_2_ treatment (Fig. 7A, fourth panel, compare lanes 4 and 2), while the phosphorylation level of ERK was decreased following H_2_O_2_ treatment in the presence of SHP-2 siRNA, compared to that of CSK and GFP siRNA-transfected cells (fourth panel, compare lanes 6 and 2, 4).

### Inhibition of SHP-2 by PTPi IV Attenuates the H_2_O_2_-induced Activation of Src-mediated ERK and FAK Activation

To verify the effects SHP-2 inhibition on Src activity in astrocytes, CRT-MG cells were pretreated with a pharmacological inhibitor of SHP-2, PTP inhibitor IV (PTPi IV), at various concentrations (0–10 µM) for 30 min, treated with or without 5 mM H_2_O_2_ for 10 min, and analyzed by immunoblotting using antibodies against p-SHP-2, SHP-2, p-Src, and Src. PTPi IV significantly reduced SHP-2 phosphorylation at Y542 and Y580 (Fig. 7B, compare lanes 2, 3, and 4), which was similar to the results of our previous studies [17]. Additionally, PTPi IV induced a dose-dependent decrease in H_2_O_2_-mediated phosphorylation of Src at Tyr 419; however, no significant change was detected for Tyr 530 phosphorylation of Src. Next, to examine whether inhibition of SHP-2 affects the Src-mediated downstream signaling pathway, immunoblotting against downstream targets such as FAK and ERK was performed. As shown in Fig. 7B, the SHP-2 inhibitor PTPi IV strongly inhibited both FAK and ERK phosphorylation.

## Discussion

In this study, we have investigated the molecular mechanism for the positive role of SHP-2 in regulating Src activity in response to H_2_O_2_-mediated oxidative stress in brain astrocytes. Our results provide initial evidence that H_2_O_2_ triggers complex formation between caveolin-1 and SHP-2, which interferes with the association between caveolin-1 and CSK and leads to enhanced activation of Src, at least in brain astrocytes.

**Figure 8:**
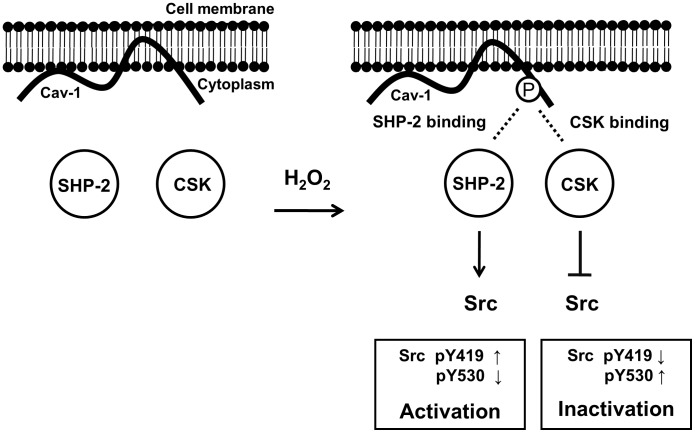
Proposed model. The binding pattern of caveolin-1, SHP-2, and CSK under oxidative stress. In astrocytes, H_2_O_2_ induces phosphorylation of caveolin-1 at Tyr 14, which provides a docking site for CSK or SHP-2. CSK binding to phosphorylated caveolin-1 induces phosphorylation of Src at Tyr 530 and inactivation of Src. In contrast, SHP-2 binding to phospho-caveolin-1 interferes with complex formation between caveolin-1 and CSK, leading to decreased phosphorylation of Src Y530 and prolonged Src activation. These results indicate that SHP-2 and CSK reciprocally regulate H_2_O_2_-induced Src activity through competitive binding to caveolin-1 in astrocytes (p, phosphor; Y, tyrosine).

Because both SHP-2 and CSK interacted with phospho-Tyr 14 of caveolin-1 (Fig. 4 and 5) and H_2_O_2_-induced Src phosphorylation was affected by SHP-2 and CSK in an inverse manner (Fig. 7), we propose that SHP-2 competitively inhibits the binding of CSK to caveolin-1 and functions as a positive regulator of the Src signaling pathway (Fig. 8). H_2_O_2_ induces phosphorylation of caveolin-1 at Tyr 14, which provides a docking site to cytoplasmic proteins, such as CSK and SHP-2. When CSK interacts with phosphorylated caveolin-1, CSK induces phosphorylation of Src Y530 to inactivate the catalytic activity of Src. However, if SHP-2 binds to phospho-caveolin-1, SHP-2 interferes with complex formation between caveolin-1 and CSK, leads to a decrease in phosphorylation of Src Y530 and an increase in phosphorylation of Src Y419, and results in sustained Src activation. These results indicate that SHP-2 and CSK reciprocally regulate H_2_O_2_-induced Src activity through competitive binding to caveolin-1 in brain astrocytes.

We observed that SHP-2 and CSK showed similar binding affinities to p-caveolin-1 (Fig. 5) and competed with each other for access to p-Y14 caveolin-1 (Fig. 6B). In our system, SHP-2 appeared to be an adaptor protein in H_2_O_2_-mediated Src signaling rather than a tyrosine phosphatase. Since SHP-2 is not a kinase, phosphorylation of Src Y419 may result from abrogating CSK action rather than inducing direct phosphorylation by SHP-2. SHP-2 is known to be either a positive or negative regulator in cellular signal transduction pathways by functioning as an adaptor protein [34]. SHP-2 exerts its negative regulatory effects through dephosphorylation or deactivation of tyrosine-phosphorylated molecules such as JAK/STATs and TrkB receptor [35], [36]. However, positive regulatory functions have also been demonstrated, as are seen in transducing signals initiated by interleukin-2 or several growth factors such as insulin, epidermal growth factor, platelet-derived growth factor, and fibroblast growth factor, which leads to activation of the MAP kinase pathway [37], [38]. Although the functions of SHP-2 in cytokine and growth factor signaling have recently been determined, the precise mechanisms by which SHP-2, a protein tyrosine phosphatase, positively regulates these intracellular signaling pathways are not well understood. In this study, we identified the putative mechanism by which SHP-2 function as an adaptor protein that competes with CSK for binding to the phosphorylated Tyr 14 residue of caveolin-1 in the presence of H_2_O_2_.

Caveolin-1 is a major multifunctional scaffolding protein of caveolae that provides a docking site to anchor various proteins, regulates a variety of signaling molecules, and modulates downstream signaling pathways negatively or positively [23], [39]. In this study, caveolin-1 acts as a docking protein for both CSK and SHP-2 in response to H_2_O_2_, suggesting that caveolin-1 may function as either positive or negative regulator in Src/ERK signaling pathways depending on the kind of binding partners. In retinal ganglion cells, caveolin-1 binds to SHP-2 enabling SHP-2-TrkB interaction, leading to TrkB receptor deactivation [36]. In brain astrocytes, SHP-2 forms complex with caveolin-1 and positively regulates Src signaling, by interfering the binding of CSK to caveolin-1. These results suggest that caveolin-1 may have distinct physiological functions in different cell types.

In both rat primary astrocytes and human astroglioma cells, H_2_O_2_ strongly induced phosphorylation of caveolin-1 at Tyr 14 (Fig. 1), which is similar to the results of previous studies performed in bovine pulmonary artery endothelial cells and placenta artery endothelial cells [28]–[30]. We found that catalase and NAC were able to suppress H_2_O_2_-induced caveolin-1 phosphorylation; however, the effect of NAC was modest compared to catalase. NAC is considered as a nonspecific ROS scavenger due to its reducing property and free radical scavenging property through increasing intracellular level of glutathione [40]. Our data demonstrate that catalase, an enzyme which directly catalyzes hydrogen peroxide, completely abrogates H_2_O_2_-induced caveolin-1 phosphorylation, thereby suggesting that the oxidative stress mediated by exogenously treated, in other hands, externally generated H_2_O_2_ directly induced caveolin-1 phosphorylation in astrocytes. Tyrosine phosphorylation at the 14th residue of caveolin-1 plays an essential role in providing a docking site to anchor various proteins, e.g., RhoA, E-cadherin, G-proteins, and CSK [23], [24], [39]. CSK, a cytoplasmic multi-domain tyrosine kinase, is recruited to the plasma membrane through its interaction with phosphorylated caveolin-1 at Tyr 14 in response to growth factors and oxidative stress. This CSK docking process enables phosphorylation of Tyr 530 in Src, which leads to inactivation of Src activity [22], [41]. Our results demonstrate that the cytoplasmic phosphatase SHP-2 behaves similar to CSK, as SHP-2 translocates and interacts with phosphorylated caveolin-1 in response to H_2_O_2_. Complex formation between SHP-2 and caveolin-1 disturbs both the binding of CSK to caveolin-1 and the negative regulation of Src by CSK. A previous report showed that SHP-2 regulates c-Src activation by controlling CSK/VE-cadherin dynamic interactions in response to vascular endothelial growth factor [42]. However, the exact mechanisms by which SHP-2 stimulates CSK dissociation have not been clarified. Here, we propose that SHP-2 is involved in the regulation of Src as an adaptor protein that competes with CSK, at least in brain astrocytes.

Interestingly, our results showed that H_2_O_2_-induced Src activation leads to phosphorylation of ERK 1/2 and FAK, and this phosphorylation was reduced in astrocytes by PTPi IV and SHP-2 siRNA (Fig. 7A and 7B). These results indicate that SHP-2 functions as an upstream signaling molecule of Src activation in H_2_O_2_-induced signal transduction. Since Src activation was shown to be involved in the activation of FAK in different types of cells, which plays an important role in focal adhesion and cell motility [43], [44], our results suggest that SHP-2 may play an important role in regulating cell motility and cell survival by modulating Src/FAK and Src/ERK signaling pathways. Previously, it was reported that CSK binds to paxillin in focal adhesions in fibroblasts and epithelial cells and triggers Src inactivation and subsequent downregulation of FAK activation [45], [46]. CSK siRNA decreased the phosphorylation of Src Y530 and dramatically increased the phosphorylation of Src Y419 following H_2_O_2_ treatment (Fig. 7A), which is in contrast to data obtained using SHP-2 siRNA. These results suggest that SHP-2 may function as a positive regulator of Src kinase, which is negatively regulated by CSK. Thus, we conclude that SHP-2 competes with CSK for binding to caveolin-1 and triggers Src activation and subsequent ERK and FAK phosphorylation. Because SHP-2 is known to be highly expressed in the CNS [13], [14] and is reported to protect neurons from neurodegeneration during oxidative stress [15], it is important to elucidate how SHP-2-mediated modulation of Src/FAK and Src/ERK signaling contributes to neuroprotection against large amounts of ROS, which are produced throughout the brain, and which downstream genes and proteins are specifically affected by these signal transduction pathways.

In summary, this study showed that SHP-2 bound to caveolin-1 and contributed to the regulation of Src activity via the competitive inhibition of CSK in the presence of H_2_O_2_ in astrocytes. Our results suggest that SHP-2 may be one of candidate targets for oxidative brain damage, as a prominent regulator of Src signaling against excessive ROS.
